# An Evaluation of the Anesthetic Properties and Serum Biochemical Response of *Ocimum* Essential Oil as a New Anesthetic Agent for Goldfish (*Carassius auratus*)

**DOI:** 10.3390/vetsci12111069

**Published:** 2025-11-07

**Authors:** Taepin Junmahasathien, Kantaporn Kheawfu, Chuda Chittasupho, Saransiri Nuanmanee, Wasana Chaisri, Surachai Pikulkaew

**Affiliations:** 1 Department of Pharmaceutical Sciences, Faculty of Pharmacy, Chiang Mai University, Chiang Mai 50200, Thailand; taepin.j@cmu.ac.th (T.J.); kantaporn.kheawfu@cmu.ac.th (K.K.); chuda.c@cmu.ac.th (C.C.); 2 Songkhla Aquatic Animal Health Research and Development Center, Department of Fisheries, Songkhla 90000, Thailand; saransirinu@fisheries.go.th; 3 Faculty of Veterinary Medicine, Chiang Mai University, Chiang Mai 50200, Thailand; wasana.ch@cmu.ac.th

**Keywords:** anesthesia, biochemical response, *Ocimum* essential oil, goldfish

## Abstract

**Simple Summary:**

In this manuscript, we present the anesthetic effects of *Ocimum tenuiflorum* L. (OTO) and *Ocimum basilicum* L. (OBO) essential oils on goldfish. The results demonstrated that both oils are effective natural anesthetics, resulting in no mortality. The serum biochemical responses indicated that the glucose and cortisol levels in the OTO group were comparatively lower than those in the fish exposed to MS-222, suggesting that OTO causes less stress in fish than exposure to other substances. These results provide essential information for developing natural anesthetics to support sustainable aquaculture development.

**Abstract:**

The objective of this research was to investigate the anesthetic properties of essential oil from *Ocimum tenuiflorum* L. (OTO) and *Ocimum basilicum* L. (OBO), which belong to the Lamiaceae family. They are used as flavoring agents, food preservatives, and traditional medicines. The serum biochemical responses were examined post-recovery to assess the impacts of anesthetics on stress levels in comet goldfish (*Carassius auratus*). The results revealed that OTO at concentrations of 80, 100, and 120 mg/L could induce deep anesthesia in 257 s, 170 s, and 136 s, and the recovery time was 293 s, 270 s, and 263 s, respectively. A greater concentration of OTO led to shortened induction times. OBO at 250 and 300 mg/L induced deep anesthesia in 212 s and 190 s, and the recovery time was significantly prolonged to 263 s and 554 s, respectively. Moreover, the effective concentrations (EC_50_) within 3 min were 85.78 mg/L (OTO) and 306.49 mg/L (OBO). The blood parameters showed that the glucose and cortisol levels in only the OTO group remained significantly lower compared to all the other treatment groups. The ALT and ALP levels were elevated in the OBO group compared to those in the OTO group. In the OBO and MS-222 groups, albumin was significantly different from that in the OTO group. Moreover, the serum calcium, phosphorus, BUN, and creatinine levels were not significantly different in any of the groups. In conclusion, OTO and OBO have been shown to be efficient natural anesthetics in goldfish, with no mortality.

## 1. Introduction

In aquaculture, particularly for fish, anesthetics are used to alleviate stress and prevent physical harm during handling, vaccination, and transportation, thereby enhancing animal welfare [1]. This involves using anesthetic agents to induce sedation and/or anesthesia in fish for many procedures [2]. A variety of chemical agents are employed for fish anesthesia, such as MS-222 (tricaine methanesulfonate), benzocaine, quinaldine, and etomidate [1]; however, some medicines are expensive or cause undesirable side effects, such as increased mucus production, hyperactivity, and gill irritation, and exhibit a limited safety margin [1,3,4]. Therefore, edible plant essential oil provides a great substitute for manufactured products. Clove essential oil from the *Syzygium aromaticum* tree is one of the most commonly used natural anesthetics employed to sedate fish [1]. Many studies have been carried out on essential oil anesthetic agents developed from the following plants: rosewood (*Aniba rosaeodora*) [5], galangal (*Alpinia galanga*) [6], Xin Yi (*Magnolia denudate)* [7], *Lippia sidoides*, *Cymbopogon citratus* [8], and *Lippia alba* [9].

Goldfish (*Carassius auratus*), a member of the Cyprinidae family, is popular among aquaculturists and hobbyists around the globe, including in Thailand, due to its symbolism, beauty, and color. Goldfish types can be classified by certain characteristics, such as their color, body shape, eyes, and fins, with the comet, fantail, lionhead, and Oranda varieties being the most common. The edible plants *Ocimum tenuiflorum* L. and *Ocimum basilicum* L. are from the genus *Ocimum*, which belongs to the Lamiaceae family, and are used as flavoring agents, food preservatives, and traditional medicines in Central Africa and Southeast Asia [10]. Due to their bioactivity, essential oils of plants of the genus *Ocimum* have a variety of pharmacological properties, displaying anticancer, antioxidant, antibacterial, anti-inflammatory, and antiparasitic activity, for example [11,12,13]. Some fish varieties have been reported to benefit from the anesthetic and sedative effects of the *Ocimum* plant, as has been shown for *Ocimum gratissimum* in Asian redtail catfish (*Hemibagrus wyckioides*) [14] and *Ocimum basilicum* in zebrafish (*Danio rerio*) [15], juvenile tambaqui (*Colossoma macropomum*) [16], Nile tilapia (*Oreochromis niloticus*), and yellowtail clownfish (*Amphiprion clarkii*) [17].

Due to the increasing need for anesthesia or sedation to control fish during handling and transportation directly reduces mortality. We selected the comet goldfish (*Carassius auratus*) as a practical, biologically relevant cyprinid model for evaluating plant essential oils as fish anesthetics, expected to be broadly informative for other freshwater cyprinids and commonly farmed species used in similar husbandry contexts. This study’s objective was to determine the anesthetic activity of *Ocimum tenuiflorum* L. and *Ocimum basilicum* L. essential oils and to analyze the serum biochemical response following induction and recovery to confirm their potential as natural anesthetics for use in ornamental fish farming, in accordance with sustainable aquaculture practices.

## 2. Materials and Methods

### 2.1. Chemical Composition of Essential Oils

The *Ocimum tenuiflorum* L. and *Ocimum basilicum* L. essential oils (OTO and OBO, respectively) used in this study were obtained from Thai-China Flavours & Fragrances Industry (Nonthaburi, Thailand). The compositions of the essential oils were analyzed using gas chromatography–mass spectrometry (GC–MS). An HP-7890A/5975C GC-MS system (Agilent Technologies, Wilmington, NC, USA) with a DB-5MS column (30 m × 0.25 mm I.D. × 0.25 µm, Agilent Technologies) was used under the following conditions: an MS transfer line heater of 280 °C and an injector temperature of 250 °C. The sample was injected using a split ratio of 1:500. The oven temperature for OTO was initially held at 60 °C for 5 min, then programmed to increase from 60 °C to 240 °C at 5 °C/min, and was finally maintained for 5 min at 240 °C. Instead, the column temperature for OBO was programmed as follows: 55 °C to 120 °C at 20 °C/min, 120 °C to 150 °C at 1.5 °C/min, 150 °C to 250 °C at 20 °C/min, and 250 °C (10 min). Helium gas was used as a carrier gas at a flow rate of 1.0 mL/min. An Agilent 5975C mass spectrometer was operated in the electron ionization mode at 70 eV with a source temperature of 230 °C, a quadrupole set to 150 °C, and a scan range from 50 to 550 *m*/*z* in the full-scan mode. Volatile compounds were identified by their mass spectra with a computerized MS database using the W8N08 library (John Wiley & Sons, Inc., Hoboken, NJ, USA).

### 2.2. Animal Maintenance and Rearing Conditions

Both juvenile (weight 9.7 ± 0.1 g and standard length 5.9 ± 0.2 cm) and adult (weight 195.4 ± 2.6 g and standard length 13.8 ± 0.4 cm) comet goldfish (*Carassius auratus*) were obtained from an ornamental fish shop in Chiang Mai, Thailand. The juvenile goldfish were used for anesthesia induction and recovery experiments (Section 2.3.1) because their smaller body size makes them more practical for handling, observing, and evaluating induction and recovery times, and the adults were used for laboratory analysis experiments (Section 2.3.2) because their larger size provides sufficient blood volume for reliable sampling. During acclimatization, the fish were stocked in a 300 L plastic tank, with dechlorinated tap water (pH 7.4–7.8; total hardness 122 mg/L; alkalinity 110 mg/L; total ammonia nitrogen and nitrite were negative) changed daily (30–50%). The fish were fed daily with a commercial pelletized diet containing 30% crude protein (Tetra Werke, Melle, Germany) and subjected to natural light conditions. According to the clinical standard examination, some fish was submitted for parasitological analysis of mucus on the skin and fin, as well as a gill scraping. Samples were examined with a light microscopy. Following a two- to four-week quarantine period, the fish were fasted for 12 h before being moved to a glass tank for the anesthesia induction and recovery experiment.

### 2.3. Experimental Design

#### 2.3.1. Anesthesia Induction and Recovery

To make the anesthetic agent, *Ocimum* essential oil was first dissolved in absolute ethanol at a ratio of 1:9 (oil-to-ethanol). The oil was then diluted further in tap water (in an anesthetic tank) until the correct concentration was reached. Ethyl 3-aminobenzoate methanesulfonate (MS-222) was purchased from Sigma-Aldrich (Wilmington, NC, USA) and used as a control. To make the MS-222 solutions (200 mg/L), MS-222 was dissolved in filtered tap water with sodium bicarbonate (Merck Millipore, Darmstadt, Germany) added at a 2:1 ratio (NaHCO_3_:MS-222) to keep the pH of the water at about 7. All anesthetic agent solutions were prepared fresh before use. We used mg/L as the concentration unit for all anesthetic agents.

A simple, completely randomized experimental design was used that included 9 groups: MS-222 (200 mg/L), OTO (40, 60, 80, 100, and 120 mg/L), and OBO (200, 250, and 300 mg/L) (each group contained 10 fish). The juvenile comet goldfish were individually placed in 1 L aquaria containing the anesthetic solution and were monitored visually with the aim of assessing the behavioral patterns for each stage of anesthesia, as described by Ross and Ross [18] (Table 1). When the surgical anesthesia stage (stage 4) was reached, verified by using the forceps response technique (caudal peduncle pinch test), the fish were immediately transferred to a recovery tank containing filtered tap water with oxygen to assess the anesthetic recovery time. After every 3 repetitions, a new anesthetic solution was made. The concentrations of *Ocimum* oil were evaluated based on induction and recovery times following a preliminary study. The maximum observation time in this investigation was 20 min. A tank containing absolute ethanol (the same as the maximum amount used in each *Ocimum* oil) in tap water was used as a vehicle control. Following their recovery, we divided the fish into groups based on their treatments to assess their clinical sign and mortality over a period of 1 week.

#### 2.3.2. Laboratory Analyses

Adult comet goldfish were fasted for 1 day before the analyses. The effect of *Ocimum* essential oil on serum biochemical response was investigated by determining serum profiles of fish after being anesthetized with OTO at 100 mg/L, OBO at 300 mg/L, and MS-222 (200 mg/L) (each group contained 10 fish). These concentrations and periods were selected in order to induce stage 4 of anesthesia around 3 min following the previous experiment (Section 2.3.1). Blood samples were obtained from the caudal vein after anesthetization using non-heparinized syringes and these were immediately transferred to non-heparinized 1.5 mL tubes. The tubes were centrifuged (3000 rpm, 10 min) in order to obtain serum, samples of which were then stored at −80 °C until further analysis [19]. Following their recovery, we divided the fish into groups based on their treatment to assess their clinical sign and mortality over a period of 1 week.

Serum concentrations of globulin, albumin, total protein, ALP, and ALT were determined in our samples using a commercial kit (DiaSys Diagnostic Systems, Rhineland-Palatinate, Germany) with an Automated Clinical Chemistry Analyzer (Sysmex BX-3010, Jln Tukang, Singapore) at a small animal hospital in the Faculty of Veterinary Medicine, Chiang Mai University. We conducted the analyses, following the manufacturer’s instructions for each run. Before performing the laboratory analysis, the machine was calibrated and validated with standards. Serum cortisol was determined using single direct antibody competitive enzyme immunoassays previously described by Brown et al. [19]. The absorbance was measured using an ELISA Reader Sunrise (Tecan, Grödig, Austria) at 405 nm, with the results expressed as ng/mL; glucose was also measured using a glucometer (Accu-Chek^®^, Roche, Melbourne, Australia).

### 2.4. Statistical Analysis

R Studio with R version 4.3.2 (2023-10-31) was used to analyze data and create graphs. The Shapiro–Wilk test was employed to evaluate the Gaussian distribution of the data, while non-parametric datasets were presented using the median and interquartile range. Differences between groups were analyzed using the Kruskal–Wallis test, followed by Wilcoxon rank sum post hoc tests with Bonferroni correction. The statistical significance was determined at *p* < 0.05. The response to anesthesia at 180 s, in the form of binomial data, was analyzed using logistic regression to determine the Half Maximal Effective Concentration (EC50), which indicates the concentration of a required chemical to elicit a 50% reaction following a defined exposure duration. This concentration is considered ideal for an anesthetic in fish.

## 3. Results

### 3.1. Chemical Composition of Essential Oils

The analysis identified methyl eugenol (28.87%), caryophyllene (28.29%), and eugenol (21.63%) as the three major constituents of OTO and estragole (72.55%) as the major constituent of OBO, with benzyl benzoate (11.80%) and linalool (10.00%) as other minor constituents. Representative GC–MS chromatograms of OTO and OBO are presented in Supplementary Figures S1 and S2, respectively. The compounds identified in OTO and OBO are listed in Table 2 and Table 3 in the order of their elution from the column, along with the percentage composition of each component and its retention index. The mass spectra of the major and minor compounds closely matched the corresponding standard library at a rate of 97–99%.

### 3.2. Anesthesia Induction and Recovery Results

Table 4 presents the induction and recovery time of the fish given OTO, OBO, and MS-222. The experiment revealed that the *Ocimum* essential oils exhibited an anesthetic effect on goldfish at different concentrations. The time to anesthesia induction for the fish was shown to decrease substantially by increasing the OTO and OBO concentrations (*p* < 0.05). OTO at a concentration of 40 mg/L was capable of inducing anesthesia stage 2 within 127 s but was incapable of inducing stages 3 and 4 during the 20 min evaluation period. At a dosage of 60 mg/L, OTO induced anesthesia stage 3 at 270 s but failed to induce stage 4 during the same evaluation period. The exposure of fish to 80–120 mg/L OTO resulted in induction times of 33–32, 45–34, 190–90, and 257–136 s to reach stages 1, 2, 3, and 4 of anesthesia, respectively.

Additionally, OBO at a concentration of 200 mg/L induced anesthesia stage 3 within 85 s; however, it did not induce stage 4 during the 20 min study period. When the fish were exposed to 250–300 mg/L OBO, the time to induce stages 1, 2, 3, and 4 of anesthesia were 51–33, 68–60, 92–82, and 212–190 s, respectively. An ethanol concentration of 0.3% *v*/*v* did not induce sedation in the fish or have any adverse effects.

**Figure 1:**
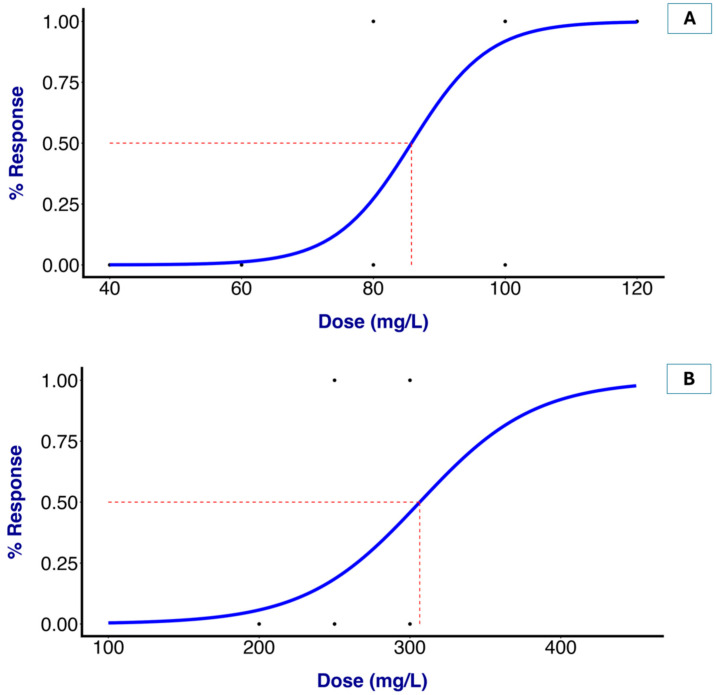
Half maximal effective concentration of *Ocimum* essential oil for an ideal anesthetic in fish. Plot of EC_50_ for OTO = 85.78 mg/L (**A**) and EC_50_ for OBO = 306.48 mg/L (**B**), leading to anesthetic stage 4 in goldfish.

In comparison with MS-222, OTO at 100 and 120 mg/L showed no difference in the induction time at stage 4 when compared to all of the examined groups. No fish mortality was observed following the administration of OTO, OBO, and MS-222 at any of the tested concentrations throughout the two-week observation period. The recovery times for the fish subjected to 80, 100, and 120 mg/L OTO were 293, 270, and 263 s, respectively. Greater concentrations of OTO led to a shortening of the recovery time, but there was no significant difference. However, the recovery times for the fish exposed to 250 and 300 mg/L OBO were 263 and 554 s, respectively. Nevertheless, higher doses of OBO led to a significantly prolonged recovery time. All of the groups demonstrated a statistically significantly increased recovery time compared to that given MS-222 (200 mg/L). After analyzing the time to stage 4 induction using the logistic regression model, the EC_50_ values for the OTO and OBO treatments were determined as 85.78 and 306.49 mg/L, respectively (Figure 1). No mortality or abnormal clinical signs were observed in any treatment group during the observation period after the experiment.

### 3.3. Laboratory Analyses

Table 5 presents the serum biochemical responses of the fish after exposure to OTO, OBO, and MS-222. For the stress indicator, the OTO group was found to have significantly lower cortisol values (81.56 ng/mL) compared to the other treatment groups, with no statistical difference identified between the cortisol levels for the fish given OBO and MS-222 (210.19 and 240.07 ng/mL, respectively). Finally, the glucose level showed the same results as the cortisol level. The glucose level in the OTO group was significantly lower (52.77 mg/dL), with no statistical difference between that in the OBO and MS-222 groups, which were 81.10 and 84.00 mg/dL, respectively.

The total concentrations of serum proteins were significantly different between all of the treatment groups, with the OTO, OBO, and MS-222 groups exhibiting values of 2.35, 2.65, and 2.90 g/dL, respectively. The albumin result showed significantly low levels in the OTO group (0.90 g/dL), while the OBO and MS-222 groups showed no difference at the same level (1.10 g/dL). While the MS-222 group had a significantly higher globulin value (1.75 g/dL), the results for the OTO and OBO groups were the same (1.50 g/dL). The OBO and MS-222 groups did not differ in their albumin-to-globulin ratio (0.60 and 0.63), but the OTO group had a significantly high value (0.69).

The ALT level of the OTO group (2.75 U/L) was significantly lower compared to that in the other experimental groups, and the ALT levels of the OBO and MS-222 groups were not significantly different (4.50 and 5.00 U/L, respectively). In addition, the ALP levels of the OTO and MS-222 groups were not significantly different (9.30 and 13.00 U/L, respectively), but the OBO group had a significantly higher ALP level than the other groups (19.50 U/L). Finally, no statistically significant differences were identified in the serum calcium and phosphorus levels or the kidney function tests (creatinine and BUN).

## 4. Discussion

As aquaculture demand increases, production intensification elevates the risk of hazardous drug and chemical residues. To mitigate economic losses, current research prioritizes safer options, notably probiotics and natural product approaches [20,21,22]. Various studies have been undertaken to assess the anesthetic effects of medicinal plants in aquatic species [5,23,24,25,26]. Numerous investigations have been conducted on anesthetizing goldfish using essential oils [5,23,27], but no information is currently available regarding the efficacy of *Ocimum* essential oil as an anesthetic in this species. In this study, the time to reach deep anesthesia exceeded 20 min for the goldfish exposed to 40 and 60 mg/L OTO, indicating that these concentrations were inadequate for achieving deep anesthesia (stage 4). Conversely, at concentrations of 80 mg/L, 100 mg/L, and 120 mg/L, this essential oil could induce deep anesthesia in 257, 170, and 136 s, with corresponding recovery times of 293, 270, and 263 s, respectively. Maryani et al. [28] also reported that OTO concentrations between 100 and 200 mg L^−1^ for administration during transportation caused total unconsciousness for about 15 min, as shown by catfish (*Pangasius* sp.) falling to the bottom of the tank and exhibiting slow operculum movements. The three main active compounds in OTO found in this study were eugenol, methyl eugenol, and caryophyllene, all known to have an anesthetic effect, especially eugenol. Eugenol and methyl eugenol, major components of clove oil, are widely used as anesthetics in fish [24]. According to previous studies, eugenols are effective at inducing anesthesia in a variety of fish species, ranging in concentration from 50 to 100 mg/L [24]; their rapid anesthesia-inducing properties permit easy fish handling and recovery [29,30]. Caryophyllene in *Aloysia triphylla* essential oil has been found to contribute significantly to its anesthetic properties, especially when used on silver catfish (*Rhamdia quelen*). However, the caryophyllene concentration in this plant is subject to seasonal variation, which impacts its anesthetic effectiveness [31]. Although eugenol (the major active compound in clove oil) is widely used as a natural anesthetic, its use may be associated with certain limitations. Clove oil has been reported to exhibit a narrow safety margin, with increased doses above the effective concentration leading to stress or mortality [32]. By comparison, *Ocimum* essential oils contain phenolic compounds such as methyl chavicol and the terpene linalool, which contribute to their antioxidant effects and may play a role in modulating stress responses during anesthesia [33,34].

The goldfish in this study were also exposed to OBO at a concentration of 200 mg/L, which was sufficient to cause stage 3 anesthesia but not stage 4. Deep anesthesia was induced by OBO concentrations of 250 and 300 mg/L in 212 and 190 s, with a recovery time of 263 and 554 s, respectively. Similarly, according to a previous study, OBO at a 300 mg L^−1^ dose was shown to be the most effective anesthetic for rainbow trout (*Oncorhynchus mykiss*) [35]. For pacu (*Piaractus mesopotamicus*), an induction time of 193 s and a recovery time of 244 s was found with 300 mg L^−1^ of OBO [36], while 400 μL L^−1^ of OBO led to the best induction and recovery times (135.2 and 199.1 s, respectively) in juvenile Nile tilapia [37]. Estragole, also known as methyl chavicol, and linalool, major compounds in OBO, have demonstrated anesthetic effects in juvenile tambaqui (*Colossoma macropomum*). These compounds can be absorbed and distributed quickly in the plasma, muscle, and brain of fish, reaching their maximum concentration within six minutes. Both methyl chavicol and linalool have a relatively short half-life and are rapidly eliminated from the body of the fish. The OBO was safe at a concentration of 800 mg/L, effectively inducing anesthesia, reducing stress responses, and permitting rapid recovery without imposing long-term histological damage on the spleen and liver [38]. Accordingly, it can be assumed from the results of this study that the anesthetic activity of *Ocimum* oil in goldfish is related to the presence of eugenol, methyl eugenol, linalool, estragole, and caryophyllene.

To be effective, a fish anesthetic should act rapidly and permit quick recovery while decreasing stress and hyperactivity. An optimal anesthetic should achieve the induction of anesthesia within three minutes, while the recovery time should be no more than twice the induction time [18]. In the present study, the concentrations (EC_50_) of OTO and OBO that effectively achieved this within three minutes were 85.78 mg/L and 306.49 mg/L, respectively. In 2012, Akbulut et al. [39] reported that the EC_50_ at three minutes for clove oil in Siberian sturgeon fry (*Acipenser baerii* Brandt, 1869) was 356 mg/L. The effective concentration of *Ocimum* oil was found to be lower than that of clove oil, but some previously documented findings revealed lower effective concentrations of clove oil in goldfish than those in the present study, such as 75–150 mg/L [27]. The efficacy of an anesthetic can be influenced by numerous factors; the response to an anesthetic varies significantly among fish species and even within the same species, which may be explained by differences in the pharmacokinetics and pharmacodynamics of different anesthetics [29,40]. Moreover, biological factors, including health status, gender, age, body weight, growth rate, and physiological state, as well as environmental variables such as water quality and water temperature, can influence the duration of induction and recovery from anesthesia [18]. Gamma-aminobutyric acid, or GABA, contributes to achieving anesthesia in fish. Natural anesthetics and chemicals such as propofol and diazepam increase GABAergic activity, producing potential synergistic effects when used in combination with other GABA-modulating substances [6,41]. Essential oil compounds such as linalool and eugenol have been shown to interact with the GABAergic system [42]. According to Meyer and Fish [43], the GABA_A_ eugenol inhibits pain perception, causing an analgesic or anesthetic effect. Fish may be anesthetized by the major component in *Ocimum* essential oil via the GABA receptor or other mechanisms, but further study is required to understand the mechanism of each constituent and the synergy between them.

For accurate diagnosis, it is essential that blood parameters be examined to provide important data on the fish’s physiological response. Hematological and biochemical profiles are commonly used to evaluate the impact of anesthetics on aquatic animals [40]. Particularly in anesthesia research, the cortisol and glucose levels in blood plasma are significant signs of stress in fish: elevated glucose levels in fish blood samples are likely to be caused by a severe response to stress induced by handling, contributing to increased energy demand thereafter [40]. The blood glucose and plasma cortisol levels of resting goldfish are approximately 43.1 ± 4.5 mg/dL and 89.7 ± 32.0 ng/mL [44]. The various anesthetics exhibited distinct effects on the blood cortisol levels. For example, the glucose and cortisol levels in goldfish exposed to OTO were found to remain relatively steady at normal levels, whereas the fish exposed to OBO and MS-222 exhibited higher blood glucose and cortisol levels than the resting fish. Factors that affect cortisol levels include the anesthetic concentration and duration of exposure. Anesthesia can alter primary (cortisol) and secondary (glucose) stress responses in fish, with direction and magnitude depending on agent, dose, exposure time, and species, complicating direct comparisons to the physiology of awake individuals [44]. Because anesthesia modulates endocrine and metabolic indicators, our conclusions are restricted to between-protocol comparisons under anesthesia rather than to non-anesthetized conditions [44]. *Salmo salar* treated with MS-222 or benzocaine exhibited elevated cortisol release rates compared to those exposed to metomidate or isoeugenol [45]. Interestingly, Silva et al. [46] found that *Ocimum americanum* essential oil effectively prevented increases in the cortisol level in *Rhamdia quelen* following exposure to air.

The elevated serum calcium and phosphorus levels post-anesthesia may be related to acute respiratory acidosis, whereas a decrease in such levels has been observed in cases of respiratory alkalosis [47]. There was no significant difference in the serum calcium and phosphorus levels among the three groups in this study. These results align with research on Vimba bream (*Vimba Vimba*), which revealed comparable levels of calcium, magnesium, and phosphorus for both the non-anesthetized control group and fish anesthetized with MS-222, clove oil, 2-phenoxyethanol, and propiscin [48]. Additionally, a study using MS-222, tobacco extract, propiscin, and clove oil in rohu (*Labeo rohita*) found no significant differences in the calcium and magnesium levels [49]. In Russian sturgeon (*Acipenser gueldenstaedtii*) anesthetized with eugenol, no significant alterations were exhibited in the calcium levels compared to the control group, whereas fish sedated with propofol showed a considerable elevation in calcium levels [50].

To evaluate the liver function of animals, the liver enzymes alanine aminotransferase (ALT) and alkaline phosphatase (ALP) are employed. An elevation in both enzymes can indicate liver injury, with ALT suggesting liver cell damage and ALP suggesting bile duct blockage or bone disease [51]. The groups anesthetized with OBO in this study showed elevated ALT and ALP levels compared to those of the OTO group, suggesting that OBO causes greater harm to the liver than OTO. Nevertheless, Lepic et al. [48] found that Vimba bream (*Vimba Vimba*) anesthetized with MS-222 and clove oil exhibited no differences in ALT, ALP, and creatine kinase when compared to non-anesthetized controls. A recent study discovered that the active compounds in essential oils, such as thymol and 1,8-cineole, did not influence the ALT levels in *Cyprinus carpio* and *Oncorhynchus mykiss* [52,53].

Due to the possible source of ALP from extrahepatic tissues and ALT can be affected by nonhepatic and periprocedural factors, these changes are nonspecific; hepatobiliary injury cannot be confirmed without histopathology [54].

In fish, elevated BUN and creatinine levels may indicate renal impairment, while BUN also indicates respiratory and excretory compromise, which may be the result of gill dysfunction [18]. Diet, water pollution, and health status may all have an effect [51]. In the present study, there were no observed changes in BUN and creatinine in any group. The plasma total protein and globulin reflect the non-specific immunity status and stress in fish exposed to various substances [16,52]. Albumin is an important blood protein that is synthesized in the liver, the level of which changes because of severe hepatic damage or hemoconcentration/hemodilution. According to the results of this study, the fish anesthetized with OTO exhibited no changes in albumin levels, consistent with the findings of Yousefi et al. [52], implying that anesthesia with 30 and 80 mgL^−1^ of thymol/eugenol does not cause liver damage or alterations in blood concentration. Under short-term stress, many proteins and peptides demonstrate elevated levels as a defensive strategy to safeguard the fish against stress and immunosuppression [55,56]; for example, plasma globulin is a sign of stress in fish. Because absolute ethanol was used only as the anesthesia vehicle and we did not examine its effect on the serum biochemical response, potential solvent confounding remains a limitation.

## 5. Conclusions

In this study, we show that OTO and OBO are efficient natural anesthetics in goldfish, and their use did not lead to mortality. A variety of substances in these essential oils possess anesthetic activity, as shown by prior reports on fish. OTO demonstrates greater benefits over OBO in reducing stress in fish during handling. Furthermore, it is possible to develop a pharmaceutical preparation of this essential oil at a precise dosage that is easy to administer to fish. More studies are needed to understand how the active ingredients in these essential oils work as anesthetics, as well as their potential cell toxicity.

## Figures and Tables

**Table 1:** Stages of anesthesia and behavior of fish.

Stage	Description of Behavior
Anesthesia	
1	Responsive to stimuli; movement reduced
2	Partial loss of equilibrium; fish rolls and attempts to right itself
3	Total loss of equilibrium; ventilation reduced
4	Failure to respond to any external stimuli
5	No ventilation; collapse and death
Recovery	Swimming resumed, balance achieved, and responsiveness

**Table 2:** Chemical constituents found in OTO.

No.	Retention Time (Rt)	Compounds	%Area	%QA
1	6.585	α-Pinene	0.35	96
2	7.138	Camphene	0.38	97
3	8.083	β-Pinene	0.22	97
4	9.868	Limonene	0.17	98
5	9.969	1,8-Cineol	0.89	98
6	12.282	Linalool	1.10	97
7	13.776	Camphor	0.40	98
8	14.308	Isoborneol	0.71	95
9	14.579	Borneol	0.79	95
10	15.297	α-Terpineol	0.40	91
11	15.381	Estragole	0.63	98
12	19.679	Camphene	0.82	94
13	19.871	Eugenol	21.63	98
14	20.488	α-Copaene	0.45	97
15	20.646	Cembrene A	0.15	80
16	20.703	β-Bourbonene	0.13	96
17	20.869	(-)-β-Elemene	6.47	99
18	21.180	Methyl eugenol	28.87	98
19	21.258	Caryophyllene	0.15	96
20	21.716	β-Caryophyllene	28.29	99
21	22.569	α-humulene	1.39	98
22	22.811	Ethyl-cinnamate	0.13	98
23	23.006	γ-Selinene	0.14	91
24	23.207	β-Cubebene	1.31	98
25	23.407	β-Selinene	0.59	99
26	23.579	α-Selinene	0.68	99
27	23.865	β-Elemene	1.25	93
28	24.111	Δ-Cadinene	0.23	99
29	24.830	Hedycaryol	0.14	83
30	25.653	β-Caryophyllene oxide	0.91	99
31	27.398	Bulnesol	0.14	93

QA = % similarity or quality match with the Wiley database.

**Table 3:** Chemical constituents found in OBO.

No.	Retention Time (Rt)	Compounds	%Area	%QA
1	4.337	trans-Linalool oxide	0.05	86
2	4.509	α-Terpinolen	0.10	96
3	4.627	Linalool	10.00	97
4	4.957	Neo-Allo-Ocimene	0.02	97
5	5.376	Dextro-camphor	0.28	98
6	5.453	(-)-Menthone	0.04	98
7	5.611	Isoborneol	0.06	80
8	5.776	L-Menthol	0.46	91
9	6.207	Estragole	72.55	98
10	6.868	(Z)-Citral	0.37	93
11	7.548	α-Citral	0.45	97
12	8.198	Isobornyl acetate	0.12	91
13	12.451	Methyl eugenol	0.53	98
14	13.721	Beta-Caryophyllene	0.41	99
15	14.454	α-Bergamotene	0.55	95
16	15.622	(E)-Beta-Farnesene	0.15	96
17	15.732	α-Caryophyllene	0.18	97
18	17.266	(E)- Germacrene D	0.24	97
19	21.228	α-Bisabolene	1.35	98
20	34.969	Benzyl benzoate	11.80	97

QA = % similarity or quality match with the Wiley database.

**Table 4:** The median (M) and interquartile range (IQR; Q1–Q3) of induction time of each stage and full recovery time of anesthetic formulations (*n* = 10).

Agent	Concentration (mg/L)	Induction Time (s)								Recovery Time (s)	
		Stage 1		Stage 2		Stage 3		Stage 4			
		M	IQR	M	IQR	M	IQR	M	IQR	M	IQR
MS-222	200	31.00 ^ab^	28.50–35.25	51.50 ^abc^	46.75–56.75	98 ^ab^	85.00–118.75	110.00 ^a^	105.75–136.75	120.50 ^a^	110.25–131.25
OTO	40	57.50 ^c^	45.50–65.25	127.00 ^d^	120.25–148.00						
	60	33.00 ^bc^	30.25–38.50	67.00 ^c^	65.00–71.75	270.00 ^bc^	223.00–280.00				
	80	30.50 ^ab^	28.25–32.75	44.50 ^ab^	41.00–46.00	190.00 ^c^	172.25–214.00	257.00 ^d^	222.25–282.00	293.50 ^b^	279.75–301.75
	100	31.50 ^b^	29.25–35.25	41.00 ^ab^	37.25–44.00	113.00 ^a^	100.00–119.75	169.50 ^ab^	144.00–174.75	270.00 ^b^	260.75–277.50
	120	24.00 ^a^	21.25–26.75	34.00 ^a^	32.00–37.75	90.00 ^ab^	73.50–107.25	135.50 ^a^	113.75–149.25	263.00 ^b^	254.25–282.75
OBO	200	32.00 ^abc^	28.00–36.25	65.00 ^abc^	48.00–76.75	84.50 ^a^	68.25–98.00				
	250	50.50 ^bc^	46.50–56.50	67.50 ^c^	61.50–74.75	92.00 ^a^	83.50–101.75	212.00 ^cd^	199.50–235.25	262.50 ^b^	234.00–272.75
	300	32.50 ^abc^	28.75–40.75	59.50 ^bc^	47.25–70.25	82.00 ^a^	74.25–94.75	190.00 ^bc^	181.25–197.50	553.50 ^c^	519.25–591.00

Note: Different superscript letters in a column (a, b, c, and d) indicate a difference (*p* < 0.05) for a Wilcoxon rank-sum test between groups.

**Table 5:** The median (M) and interquartile range (IQR; Q1–Q3) of blood parameters of each anesthetic formulation (*n* = 10).

Blood Parameter (Unit)		Chemicals		*p-*Value (Kruskal–Wallis Test)
	OTO Median (IQR)	OBO Median (IQR)	MS-222 Median (IQR)	
Cortisol (ng/mL)	81.56 ^a^ (74.26–89.09)	210.19 ^b^ (190.79–234.16)	240.07 ^b^ (214.29–257.71)	0.00041
Glucose (mg/dL)	52.77 ^a^ (52.00–55.48)	81.10 ^b^ (75.00–86.97)	84.00 ^b^ (74.55–91.06)	0.00042
Total protein (g/dL)	2.35 ^a^ (2.28–2.40)	2.65 ^b^ (2.50–2.70)	2.90 ^c^ (2.88–2.93)	0.00008
Albumin (g/dL)	0.90 ^a^ (0.80–0.90)	1.10 ^b^ (1.00–1.10)	1.10 ^b^ (1.08–1.10)	0.00061
Globulin (g/dL)	1.50 ^a^ (1.40–1.50)	1.50 ^a^ (1.50–1.60)	1.75 ^b^ (1.60–1.83)	0.00179
ALT (U/L)	2.75 ^a^ (2.00–3.00)	4.50 ^b^ (4.00–5.63)	5.00 ^b^ (4.00–5.25)	0.00479
ALP (U/L)	9.30 ^a^ (7.63–11.63)	19.50 ^b^ (17.50–20.50)	13.00 ^a^ (11.50–14.25)	0.00028
BUN (mg/dL)	1.80 ^a^ (1.28–1.80)	1.50 ^a^ (1.28–1.80)	1.45 ^a^ (1.30–1.63)	0.98300
Creatinine (mg/dL)	0.16 ^a^ (0.12–0.25)	0.18 ^a^ (0.12–0.29)	0.49 ^a^ (0.15–0.58)	0.10800
Calcium (mmol/L)	9.35 ^a^ (8.90–10.35)	10.70 ^a^ (10.20–11.60)	10.45 ^a^ (10.15–11.28)	0.06160
Phosphorus (mg/dL)	10.00 ^a^ (9.48–11.00)	10.65 ^a^ (9.95–11.18)	10.45 ^a^ (9.95–11.15)	0.66900

Note: Different superscript letters in a column (a, b, and c) indicate a difference (*p* < 0.05) for a Wilcoxon rank-sum test between groups.

## Data Availability

The original contributions presented in this study are included in this article/Supplementary Materials. Further inquiries can be directed to the corresponding author.

## Supplementary Materials

The following supporting information can be downloaded at: https://www.mdpi.com/article/10.3390/vetsci12111069/s1, Figure S1: GC–MS chromatogram of OTO. The major peaks correspond to eugenol (Rt = 19.871 min, 21.63%), methyl eugenol (Rt = 21.180 min, 28.87%), and β-caryophyllene (Rt = 21.716 min, 28.29%); Figure S2: GC–MS chromatogram of OBO. Major peaks correspond to estragole (Rt = 6.207 min, 72.55%), benzyl benzoate (Rt = 34.969 min, 11.80%), and linalool (Rt = 4.627 min, 10.00%).

## References

[1] Martins T., Valentim A., Pereira N., Antunes L.M. (2019). Anaesthetics and Analgesics Used in Adult Fish for Research: A review. Lab. Anim..

[2] Rairat T., Chi Y., Hsieh C.Y., Liu Y.K., Chuchird N., Chou C.C. (2021). Determination of Optimal Doses and Minimum Effective Concentrations of Tricaine Methanesulfonate, 2-Phenoxyethanol and Eugenol for Laboratory Managements in Nile Tilapia (*Oreochromis niloticus*). Animals.

[3] Zhuang Z., Li X., Luo Y., Li Y., Ahmed Isse S., Zhang Z., Luo Q., Chen X. (2025). Developmental Neurotoxicity of Anesthetic Etomidate in Zebrafish Larvae: Alterations in Motor Function, Neurotransmitter Signaling, and Lipid Metabolism. J. Hazard. Mater..

[4] Neiffer D.L., Stamper M.A. (2009). Fish Sedation, Analgesia, Anesthesia, and Euthanasia: Considerations, Methods, and Types of Drugs. ILAR J..

[5] Kizak V., Can E., Danabaş D., Can S.S. (2018). Evaluation of Anesthetic Potential of Rosewood (*Aniba rosaeodora*) Oil as a New Anesthetic Agent for Goldfish (*Carassius auratus*). Aquaculture.

[6] Khumpirapang N., Chaichit S., Jiranusornkul S., Pikulkaew S., Müllertz A., Okonogi S. (2018). In Vivo Anesthetic Effect and Mechanism of Action of Active Compounds from *Alpinia galanga* Oil on *Cyprinus carpio* (koi carp). Aquaculture.

[7] Zeng X., Dong H., Zhang J., Wang W., Duan Y., Chen J., Zhang J. (2022). Essential Oil of *Magnolia denudata* is an Effective Anesthetic for Spotted Seabass (*Lateolabrax maculatus*): A Test of its Effect on Blood Biochemistry, Physiology, and Gill Morphology. Fish Physiol. Biochem..

[8] de Oliveira I.C., Oliveira R.S.M., Lemos C.H.D.P., de Oliveira C.P.B., Silva A.F., Lorenzo V.P., Lima A.O., da Cruz A.L., Copatti C.E. (2022). Essential Oils from *Cymbopogon citratus* and *Lippia sidoides* in the Anesthetic Induction and Transport of Ornamental Fish *Pterophyllum scalare*. Fish Physiol. Biochem..

[9] Toni C., Martos-Sitcha J.A., Baldisserotto B., Heinzmann B.M., de Lima Silva L., Martínez-Rodríguez G., Mancera J.M. (2015). Sedative Effect of 2-Phenoxyethanol and Essential Oil of *Lippia alba* on Stress Response in Gilthead Sea Bream (*Sparus aurata*). Res. Vet. Sci..

[10] Bristol D.W. (2011). NTP 3-Month Toxicity Studies of Estragole (CAS No. 140-67-0) Administered by Gavage to F344/N Rats and B6C3F1 Mice. Toxic. Rep. Ser..

[11] Dharsono H.D.A., Putri S.A., Kurnia D., Dudi D., Satari M.H. (2022). *Ocimum* Species: A Review on Chemical Constituents and Antibacterial Activity. Molecules.

[12] Devi P.U., Ganasoundari A. (1999). Modulation of Glutathione and Antioxidant Enzymes by *Ocimum sanctum* and its Role in Protection Against Radiation Injury. Indian J. Exp. Biol..

[13] Sharma P., Kulshreshtha S., Sharma A.L. (1998). Anti-Cataract Activity of *Ocimum sanctum* on Experimental Cataract. Indian J. Pharmacol..

[14] Lam P.H., Vo H.D.N., Truong L.M.T., Dang D.M.T., Dang C.M., Doan T.C.D., Mollaamin F., Monajjemi M. (2025). Anesthetic Effects of Clove Basil Essential Oil (*Ocimum gratissimum*) Microemulsion on Asian Redtail Catfish (*Hemibagrus wyckioides*) and Its Biochemical Stress Indicators. Fishes.

[15] Capparucci F., De Benedetto G., Natale S., Pecoraro R., Iaria C., Marino F. (2022). Evaluation of Anaesthetic Effect of Commercial Basil *Ocimum basilicum* on Zebrafish (*Danio rerio*) Embryos. Fishes.

[16] Ventura A.S., Jerônimo G.T., Corrêa Filho R.A.C., Souza A.I.D., Stringhetta G.R., Cruz M.G.D., Torres G.D.S., Gonçalves L.U., Povh J.A. (2021). *Ocimum basilicum* Essential Oil as An Anesthetic for Tambaqui *Colossoma macropomum*: Hematological, Biochemical, Non-specific Immune Parameters and Energy Metabolism. Aquaculture.

[17] Correia A.M., Pedrazzani A.S., Mendonça R.C., Massucatto A., Ozório R.A., Tsuzuki M.Y. (2018). Basil, Tea Tree and Clove Essential Oils as Analgesics and Anesthetics in *Amphiprion clarkii* (Bennett, 1830). Braz. J. Biol..

[18] Ross L.G., Ross B.R. (2008). Anesthetic and Sedative Techniques for Aquatic Animals.

[19] Brown J.L., Bansiddhi P., Khonmee J., Thitaram C. (2020). Commonalities in Management and Husbandry Factors Important for Health and Welfare of Captive Elephants in North America and Thailand. Animals.

[20] Abbas M.A., Kim H.-J., Lee G.-Y., Cho H.-Y., Al Jawad Sayem S., Lee E.-B., Lee S.-J., Park S.-C. (2025). Development and Application of *Lactobacillus plantarum* PSCPL13 Probiotics in Olive Flounder (*Paralichthys olivaceus*) Farming. Microorganisms.

[21] Jiang Y.-H., Liang M., Yang Y.-H., Xue J., Suo H.-Y. (2024). Probiotic *Lactobacillus plantarum* SHY21-2 Protected Zebrafish Against *Aeromonas hydrophila* Infection by Naintaining Intestinal Barrier Integrity, Inhibiting Inflammatory and Oxidative Stress Responses, and Regulating Intestinal Microbiome. Aquaculture.

[22] Jeong H.S., Hwang S.D., Won K.M., Hwang J.-A. (2025). Dietary Soy Isoflavones Promote Feminization and Enhance Growth of Juvenile Japanese Eel (*Anguilla japonica*). Animals.

[23] Karim A., Shahzad M.M., Kamal K., Khwaja S., Ijaz A., Imtiaz S. (2024). Efficacy of Clove Oil and Rosewood Oil as Anesthetics on Goldfish (*Carassius auratus*). Egypt. J. Aquat. Res..

[24] Ventura A.S., Jerônimo G.T., de Oliveira S.N., de Araújo Gabriel A.M., Cardoso C.A.L., Teodoro G.C., Corrêa Filho R.A.C., Povh J.A. (2020). Natural Anesthetics in the Transport of Nile Tilapia: Hematological and Biochemical Responses and Residual Concentration in the Fillet. Aquaculture.

[25] Jerez-Cepa I., Fernández-Castro M., Alameda-López M., González-Manzano G., Mancera J.M., Ruiz-Jarabo I. (2021). Transport and Recovery of Gilthead Seabream (*Sparus aurata* L.) Sedated with AQUI-S^®^ and Etomidate: Effects on Intermediary Metabolism and Osmoregulation. Aquaculture.

[26] Brandão F.R., Duncan W.P., Farias C.F.S., de Melo Souza D.C., de Oliveira M.I.B., Rocha M.J.S., Monteiro P.C., Majolo C., Chaves F.C.M., de Almeida O’Sullivan F.L. (2022). Essential Oils of *Lippia sidoides* and *Mentha piperita* as Reducers of Stress During the Transport of *Colossoma macropomum*. Aquaculture.

[27] Perdikaris C., Nathanailides C., Gouva E., Gabriel U.U., Bitchava K., Athanasopoulou F., Paschou A., Paschos I. (2010). Size-relative Effectiveness of Clove Oil as an Anaesthetic for Rainbow Trout (*Oncorhynchus mykiss* Walbaum, 1792) and Goldfish (*Carassius auratus* Linnaeus, 1758). Acta Vet. Brno..

[28] Maryani, Monalisa S.S., Rozik M., Yulintine, Dangeubun J.L., Rosdiana. (2022). Anesthesia Application of Holy Basil (*Ocimum tenuiflorum*) Leaf Essential Oil on Catfish (*Pangasius* sp.) Seed Transportation. AACL Bioflux.

[29] Kheawfu K., Pikulkaew S., Wellendorph P., Jørgensen L.G., Rades T., Müllertz A., Okonogi S. (2022). Elucidating Pathway and Anesthetic Mechanism of Action of Clove Oil Nanoformulations in Fish. Pharmaceutics.

[30] Nuanmanee S., Sriwanayos P., Boonyo K., Chaisri W., Saengsitthisak B., Tajai P., Pikulkaew S. (2024). Synergistic Effect between Eugenol and 1,8-Cineole on Anesthesia in Guppy Fish (*Poecilia reticulata*). Vet. Sci..

[31] Parodi T.V., Gressler L.T., Silva L.D.L., Becker A.G., Schmidt D., Caron B.O., Heinzmann B.M., Baldisserotto B. (2020). Chemical Composition of The Essential Oil of *Aloysia triphylla* Under Seasonal Influence and its Anaesthetic Activity in Fish. Aquac. Res..

[32] Javahery S., Nekoubin H., Moradlu A.H. (2012). Effect of Anaesthesia with Clove Oil in Fish (Review). Fish Physiol. Biochem..

[33] Zhakipbekov K., Turgumbayeva A., Akhelova S., Bekmuratova K., Blinova O., Utegenova G., Shertaeva K., Sadykov N., Tastambek K., Saginbazarova A. (2024). Antimicrobial and Other Pharmacological Properties of *Ocimum basilicum*, *Lamiaceae*. Molecules.

[34] Bakkali F., Averbeck S., Averbeck D., Idaomar M. (2008). Biological Effects of Essential Oils—A Review. Food Chem. Toxicol..

[35] Yigit N.O., Metin S., Sabuncu O.F., Didinen B.I., Didinen H., Ozmen O., Koskan O. (2022). Efficiency of *Ocimum basilicum* and *Eucalyptus globulus* Essential Oils on Anesthesia and Histopathology of Rainbow Trout, *Oncorhynchus mykiss*. J. World. Aquac. Soc..

[36] Ventura A.S., Corrêa Filho R.A.C., Cardoso C.A.L., Stringhetta G.R., de Oliveira Brasileiro L., Ribeiro J.S., Pereira S.A., Jerônimo G.T., Povh J.A. (2024). *Ocimum basilicum* Essential Oil in Pacu *Piaractus mesopotamicus*: Anesthetic Efficacy, Distribution, and Depletion in Different Tissues. Vet. Res. Commun..

[37] Netto J.D.L., Oliveira R.S.M., Copatti C.E. (2017). Efficiency of Essential Oils of *Ocimum basilicum* and *Cymbopogum flexuosus* in The Sedation and Anesthesia of Nile Tilapia Juveniles. An. Acad. Bras. Cienc..

[38] Farias C.F.S., Ventura A.S., Jerônimo G.T., Cardoso C.A.L., de Matos L.V., da Silva G.S., Gonçalves L.U., Povh J.A., Martins M.L. (2024). Pharmacokinetics and Metabolism of Basil (*Ocimum basilicum*) Essential Oil as An Anesthetic for Tambaqui (*Colossoma macropomum*). Aquac. Int..

[39] Akbulut B., Çakmak E., Özel O.T., Dülger N. (2012). Effect of Anaesthesia with Clove Oil and Benzocaine on Feed Intake in Siberian Sturgeon (*Acipenser baerii* Brandt, 1869). Turk. J. Fish Aquat. Sci..

[40] Zahl I.H., Samuelsen O., Kiessling A. (2012). Anaesthesia of farmed fish: Implications for Welfare. Fish Physiol. Biochem..

[41] Heldwein C.G., Silva L.L., Reckziegel P., Barros F.M., Bürger M.E., Baldisserotto B., Mallmann C.A., Schmidt D., Caron B.O., Heinzmann B.M. (2012). Participation of the GABAergic System in the Anesthetic Effect of *Lippia alba* (Mill.) N.E. Brown Essential Oil. Braz. J. Med. Biol. Res..

[42] Bianchini A.E., Garlet Q.I., da Cunha J.A., Bandeira G., Junior Brusque I.C.M., Salbego J., Heinzmann B.M., Baldisserotto B. (2017). Monoterpenoids (Thymol, Carvacrol and S-(+)-Linalool) with Anesthetic Activity in Silver Catfish (*Rhamdia quelen*): Evaluation of Acetylcholinesterase and GABAergic Activity. Braz. J. Med. Biol. Res..

[43] Meyer R.E., Fish R.E., Fish R.E., Danneman P.J., Brown M.J., Karas A.Z. (2008). Pharmacology of Injectable Anesthetics, Sedatives, and Tranquilizers. Anesthesia and Analgesia in Laboratory Animals.

[44] Smith M.E., Kane A.S., Popper A.N. (2004). Noise-induced Stress Response and Hearing Loss in Goldfish (*Carassius auratus*). J. Exp. Biol..

[45] Zahl I.H., Kiessling A., Samuelsen O.B., Olsen R.E. (2010). Anesthesia Induces Stress in Atlantic Salmon (*Salmo salar*), Atlantic Cod (*Gadus morhua*) and Atlantic Halibut (*Hippoglossus hippoglossus*). Fish Physiol. Biochem..

[46] Silva L.L., Garlet Q.I., Koakoski G., Abreu M.S., Mallmann C.A., Baldisserotto B., Barcellos L.J.G., Heinzmann B.M. (2015). Anesthetic Activity of the Essential Oil of *Ocimum americanum* in *Rhamdia quelen* (Quoy & Gaimard, 1824) and its Effects on Stress Parameters. Neotrop. Ichthyol..

[47] Ghosh A.K., Joshi S.R. (2008). Disorders of Calcium, Phosphorus and Magnesium Metabolism. J. Assoc. Physician. India.

[48] Lepic P., Stara A., Turek J., Kozak P., Velisek J. (2014). The Effects of Four Anaesthetics on Haematological and Blood Biochemical Profiles in Vimba Bream, *Vimba vimba*. Vet. Med-Czech..

[49] Habib S.S., Naz S., Batool A.I., Rehman M.F.U., Ullah M., Kesbiç O.S., Maricchiolo G., Fazio F. (2024). Effect of Different Anaesthetics on Hematology and Blood Biochemistry of *Labeo rohita*. Aquac. Stud..

[50] Gomułka P., Dągowski J., Własow T., Szczepkowski M., Czerniak E., Ziomek E., Szczerbowski A., Łuczyński M., Szkudlarek M. (2015). Haematological and Biochemical Blood Profile in Russian Sturgeon Following Propofol and Eugenol Anaesthesia. Turk. J. Fish Aquat. Sci..

[51] Bojarski B., Witeska M., Kondera E. (2025). Blood Biochemical Biomarkers in Fish Toxicology—A Review. Animals.

[52] Yousefi M., Hoseini S.M., Vatnikov Y.A., Nikishov A.A., Kulikov E.V. (2018). Thymol as A New Anesthetic in Common Carp (*Cyprinus carpio*): Efficacy and Physiological Effects in Comparison with Eugenol. Aquaculture.

[53] Taheri-Mirghaed A., Ghelichpour M., Zargari A., Yousefi M. (2018). Anaesthetic Efficacy and Biochemical Effects of 1,8-cineole in Rainbow Trout (*Oncorhynchus mykiss*, Walbaum, 1792). Aquac. Res..

[54] Rożyński M., Demska-Zakęś K., Sikora A., Zakęś Z. (2018). Impact of Inducing General Anesthesia with Propiscin (Etomidate) on the Physiology and Health of European Perch (*Perca fluviatilis* L.). Fish Physiol. Biochem..

[55] Caipang C.M.A., Berg I., Brinchmann M.F., Kiron V. (2009). Short-term Crowding Stress in Atlantic Cod, *Gadus morhua* L. Modulates the Humoral Immune Response. Aquaculture.

[56] Jia Y., Wang J., Gao Y., Huang B. (2021). Hypoxia Tolerance, Hematological, and Biochemical Response in Juvenile Turbot (*Scophthalmus maximus*. L.). Aquaculture.

