# Supplementary material for: An Evaluation of the Anesthetic Properties and Serum Biochemical Response of Ocimum Essential Oil as a New Anesthetic Agent for Goldfish (Carassius auratus)
Source: Vet Sci. 2025 Nov 7;12(11):1069. doi: 10.3390/vetsci12111069 (PMC12656775; doi:10.3390/vetsci12111069)
Supplement: Supplementary file 1 [file vetsci-12-01069-s001.zip › vetsci-3898475-supplementary.pdf]

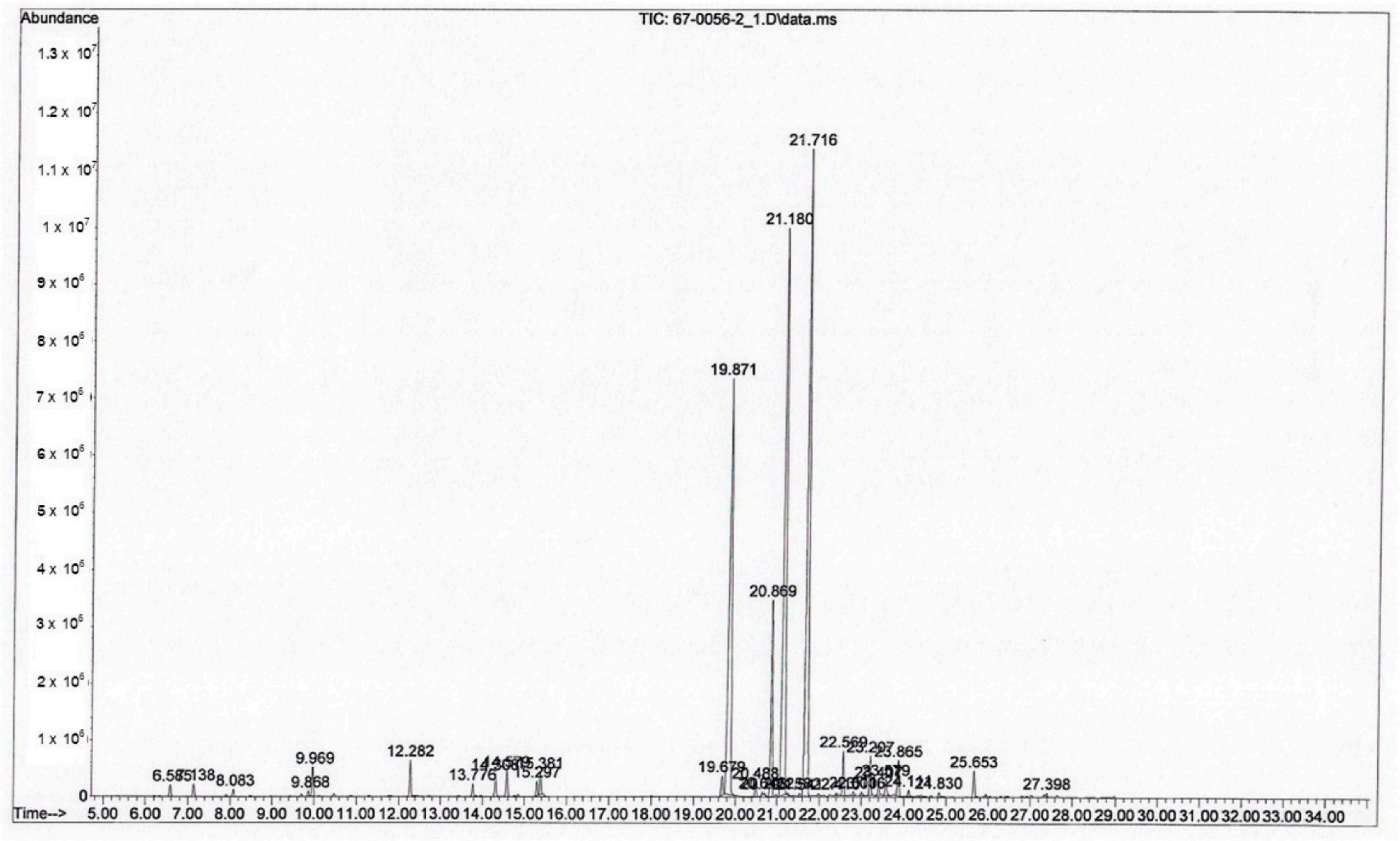


**Supplementary Figure S1.** GC–MS chromatogram of OTO. The major peaks correspond to eugenol (Rt = $19.87_1$ min, 21.63%), methyl eugenol (Rt = $21.18_0$ min, 28.87%), and β-caryophyllene (Rt = 21.716 min, 28.29%).

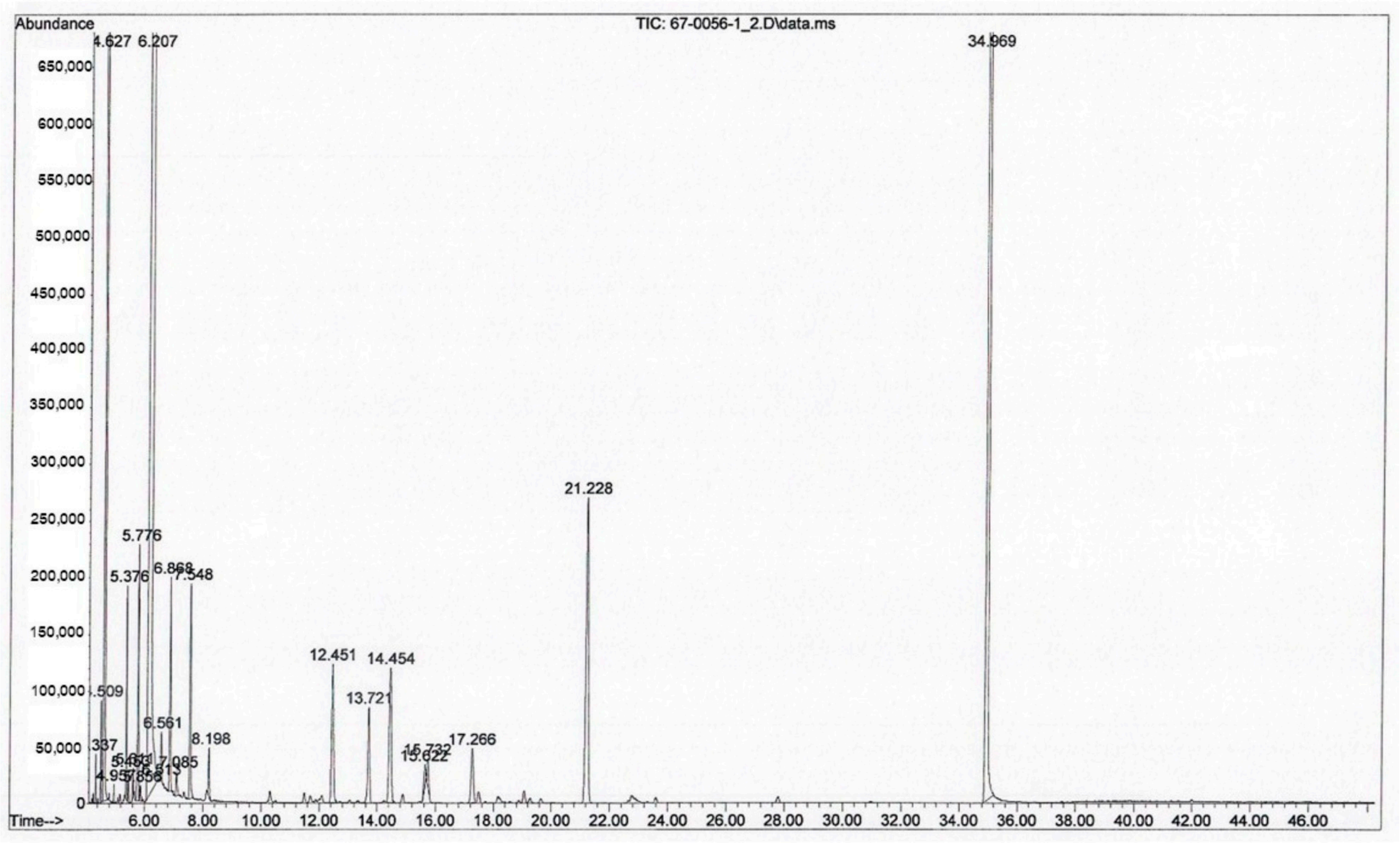


**Supplementary Figure S2.** GC–MS chromatogram of OBO. Major peaks correspond to estragole (Rt = 6.207 min, 72.55%), benzyl benzoate (Rt = 34.969 min, 11.80%), and linalool (Rt = 4.627 min, 10.00%).
